# Clinical Findings, Radiological Characteristics, and Treatment Options of Spontaneous and Secondary Intracranial Hypotension: A Single-Center Experience in Turkey

**DOI:** 10.7759/cureus.67439

**Published:** 2024-08-21

**Authors:** Aslı Yaman Kula, Saniye Karabudak

**Affiliations:** 1 Neurology, Bezmialem Foundation University, Istanbul, TUR

**Keywords:** headache, cerebrospinal fluid leakage, epidural blood patch, secondary intracranial hypotension, spontaneous intracranial hypotension

## Abstract

Introduction

Intracranial hypotension can occur for many reasons, including trauma, surgery, congenital defects, or spontaneous rupture of the dura mater. Symptoms appear long before cerebrospinal fluid (CSF) leaks are diagnosed. Treatment procedures include a variety of conservative and invasive techniques appropriate to the nature of the etiological cause and the severity of the disease. In this cross-sectional study, we aimed to investigate the clinical and imaging features and treatment options of intracranial hypotension patients and to compare them in terms of different etiologies.

Methods

The data from intracranial hypotension patients were analyzed retrospectively. Symptomatology, neurological findings, and radiological features were compared between patients with spontaneous intracranial hypotension (SIH) and those with secondary causes. Radiological outcomes of conservative treatment and epidural blood patch (EBP) were also evaluated for both groups.

Results

Of the 30 patients, 23 were female. In 14 of the patients (46.6%), a possible cause of CSF leakage was detected. Compared to intracranial hypotension patients with a secondary cause, SIH patients complained of posterior neck and shoulder pain more frequently (p=0.014, p=0.006). MRI features did not differ significantly when the two groups were compared (p>0.05). The first and sixth-month follow-up MRIs of patients treated with EBP or a conservative approach showed similar improvement rates (p=0.788).

Conclusions

There was no significant difference in radiological recovery time between conservative treatment and EBP in patients with intracranial hypotension. Radiological recovery times are similar in patients with secondary intracranial hypotension and SIH.

## Introduction

Intracranial hypotension is defined as a cerebrospinal fluid (CSF) pressure below 60 mm H_2_O, although in more than 50% of cases, the pressure exceeds 60 mm H_2_O [1]. A decline in CSF volume is believed to be responsible for this condition. Weak dura mater structure may play a role in the pathogenesis. Women are more likely to develop the disease, and it can occur in anyone at any age but is usually more common around the age of 40 [2]. Although orthostatic headache is the most common presenting complaint, patients may also have other symptoms, such as nausea and visual disturbances. They may even present with more severe symptoms, such as impaired cognition and consciousness [3].

CSF leakage may occur spontaneously or idiopathically or may develop after trauma or surgery or due to congenital defects. CSF leaks can occur in the head region, from the skull base, nasal cavity, and sinuses [4]. Non-surgical trauma causes around 80% of the rhinorrhea-related CSF fistulae, surgical trauma causes 16%, and the rest are of non-traumatic etiology [5]. In the case of spontaneous Intracranial hypotension (SIH), the spine (not the skull base) should be investigated for underlying CSF leaks [6]. Cranial magnetic resonance imaging (MRI) diagnoses intracranial hypotension in approximately 80% of cases [7]. Initial findings include subdural fluid collections, gadolinium enhancement of the pachymeninges, distension of venous structures, pituitary enlargement, and downward displacement of the brain [8-11].

Different procedures are used in the management of patients with intracranial hypotension. Conservative treatment consists of abundant hydration, bed rest, and caffeine intake. This treatment is effective in many patients [12]. In addition, epidural blood patch (EBP) therapy is beneficial in patients with CSF leakage. This option is considered after the diagnosis of intracranial hypotension is confirmed by cranial MRI, even if the site of the CSF leak has not yet been identified [13]. When clinical suspicion remains high, EBP may be recommended even in the absence of cranial MRI findings, as imaging findings may not be present in up to 20% of cases of intracranial hypotension initially [7].

Most studies have addressed SIH and secondary CSF leaks separately, but these entities are diagnosed using the same methods and treated similarly. However, the effectiveness of the conservative method and EBP is an area of ​​ongoing research. There is an insufficient number of studies comparing these treatment methods in the literature. In this study, we aimed to compare the clinical and imaging characteristics of patients who developed SIH or intracranial hypotension due to an underlying cause and to compare the radiological recovery times of patients who underwent EBP and those who did not undergo EBP.

## Materials and methods

Design

This retrospective, cross-sectional study was approved by the Institutional Review Board of Bezmialem Foundation University Medical Faculty, Turkey (No: E-54022451-050.04-149082 Date: 26.04.2024) and followed the ethical standards of the Declaration of Helsinki.

Patients

The data of 30 intracranial hypotension patients who were followed up in the Neurology Outpatient Clinic of Bezmialem Foundation University Medical Faculty Hospital between January 2018 and January 2024 and their cranial MRI examinations were analyzed. The diagnosis of low CSF pressure headache, SIH, and CSF fistula headache is based on the International Classification of Headache Disorders, third edition (ICHD-III) criteria [14]. Patients had to be older than 18 years of age, have a new persistent orthostatic headache and/or low CSF opening pressure (e.g., <6.0 cm H_2_O), and/or have positive imaging findings and/or have a known cause of CSF leakage (lumbar puncture, significant trauma, and surgery) to be included in the study. Patients younger than 18 and those who did not have an MRI at admission or whose MRI sequences were insufficient were excluded from the study.

Data collection and comparisons

Demographics (age, sex, history of hypertension (HT), history of hyperlipidemia (HL), history of diabetes mellitus (DM), history of malignancy, and infection), symptoms at presentation (headache, posterior neck pain, shoulder pain, change in hearing, tinnitus, dizziness, vertigo, nausea/vomiting, diplopia, phonophobia, photophobia, and cognitive difficulties), neurological examination findings (neck stiffness, cranial neuropathy, motor deficit, sensory deficit, and ataxia), MRI findings at presentation (diffuse meningeal thickening/enhancement, subdural collections, engorgement of the pituitary gland, inferior displacement of the midbrain, prominence of the venous sinuses, cerebellar tonsillar descent, medial and inferior migration of the medial temporal lobes) were recorded. Clinical responses, imaging findings, and treatment options were analyzed for each patient at the first and/or sixth-month follow-up.

Patients with SIH and intracranial hypotension due to an underlying cause were evaluated separately in terms of symptomatology, neurologic findings, and radiologic features. Patients treated with a conservative approach and those who underwent EBP were evaluated independently according to radiologic follow-up. Response to treatment was defined as improvement in clinical symptoms to the extent that no further treatment was required for at least six months.

Statistical analysis

We performed all statistical analyses using the IBM SPSS Statistics 26 software package (IBM, Armonk, NY, USA). For categorical variables, we report the frequency and percentage. If not normally distributed, continuous variables are reported as mean ± standard deviation or as the median and interquartile range (IQR). We checked for normality using the Shapiro-Wilk test. When Fisher’s exact test was appropriate, we used it to compare the qualitative variables. We used the independent t-test (for normal distributions) or the Mann-Whitney U test (for non-normal distributions) to compare the two groups. We report a significance level of p<0.05.

## Results

Twenty-three (74.2%) of the 30 patients were female. The mean age of the patients was 41.9±12.94 years. HT was present in 13 (43.3%) patients, HL in five (16.7%), DM in four (13.3%), and malignancy in three (10 %). Five (16.7%) patients had a pulmonary or urinary tract infection history within one month before presentation. No identifiable cause of intracranial hypotension was found in 16 (53.3%) patients, and these patients were diagnosed with SIH. There was a possible cause of CSF leakage in 14 (46.6%) patients. Intracranial hypotension occurred after trauma in two (6.7%), lumbar puncture in five (16.7%), vigorous exercise in one (3.3%), spinal anesthesia in four (13.3%), and intracranial shunt placement in two (6.7%). When the demographic characteristics of patients with SIH and intracranial hypotension due to secondary causes were compared, no significant difference was observed between the groups. Demographic characteristics of patients with SIH and intracranial hypotension due to secondary causes are presented in Table 1.

**Table 1:** Demographic characteristics of patients with SIH and CSF leakage due to secondary causes A significance level is p<0.05. ^a^Independent-sample t-test ^b^Fisher’s exact test SIH, spontaneous intracranial hypotension; CSF, cerebrospinal fluid; HT, hypertension; HL, hyperlipidemia; DM, diabetes mellitus; SD, standard deviation

Demographic characteristics	SIH (n=16)	Secondary CSF leaks (n=14)		p
Age in years, mean (SD)	45.19 (10.38)	38.14 (14.84)		0.139^a^
Female sex, n (%)	11 (68.7)	12 (85.7)		0,256^b^
HT, n (%)	9 (56.2)	4 (28.5)		0,123^b^
HL, n (%)	3 (18.7)	2 (14.2)		0.567^b^
DM, n (%)	3 (18.7)	1 (7.1)		0.352^b^
Malignancy, n (%)	2 (12.5)	1 (7.1)		0.552^b^
History of infection, n (%)	3 (18.7)	2 (14.2)		0.567^b^

Headache was absent in only four patients. Twenty-five (83.3%) patients had a postural headache that intensified when standing up and decreased when lying down. One (3.3%) patient had a sudden onset, severe, non-positional, throbbing headache requiring emergency admission. Sixteen (53.3%) patients had neck pain. Pain in the shoulders was present in seven (23.3%) patients. Nausea and vomiting were present in 19 (63.3%) patients, decreased hearing in four (13.3%), tinnitus in 16 (53.3%), dizziness in 16 (53.3%), and vertigo in four (13.3%). Seven (23.3%) patients had diplopia, 11 (36.7%) had photophobia, and five (16.7%) had phonophobia. Only two (6.7%) patients complained of impaired cognitive functions. Neurologic examination revealed neck stiffness in six (20%) patients, superficial sensory deficit in four (13.3%) patients, and ataxia in three (10%) patients. No cranial neuropathy was observed in any patient. Motor system examination was normal in all patients. When the clinical symptoms and examination findings of patients with SIH and intracranial hypotension due to secondary causes were compared, posterior neck pain and shoulder pain were significantly more common in SIH patients (p=0.014, p=0.006). Clinical symptoms and examination findings of patients with SIH and intracranial hypotension due to secondary causes are presented in Table 2.

**Table 2:** Clinical characteristics of patients with SIH and CSF leakage due to secondary causes A significance level is p<0.05. ^a^Fisher’s exact test SIH, spontaneous intracranial hypotension; CSF, cerebrospinal fluid

Clinical manifestations	SIH (n=16)	Secondary CSF leaks (n=14)	p^a^
Headache, n (%)	14 (87.5)	12 (85.7)	0.648
Orthostatic headache	13 (81.2)	12 (85.7)	
Non-orthostatic headache	1 (6.2)	0	
Posterior neck pain, n (%)	12 (75)	4 (28.5)	0.014
Shoulder pain, n (%)	7 (43.7)	0	0.006
Change in hearing, n (%)	2 (12.5)	2 (14.2)	0.648
Tinnitus, n (%)	7 (43.7)	9 (64.2)	0.225
Dizziness, n (%)	7 (43.7)	9 (64.2)	0.225
Vertigo, n (%)	1 (6.2)	3 (21.4)	0.249
Nausea/vomiting, n (%)	10 (62.5)	9 (64.2)	0.610
Diplopia, n (%)	2 (12.5)	5 (35.7)	0.143
Phonophobia, n (%)	3 (18.7)	2 (14.2)	0.567
Photophobia, n (%)	7 (43.7)	4 (28.5)	0.317
Cognitive difficulties	2 (12.5)	0	0.276
Neurological examination			
Neck stiffness, n (%)	5 (31.2)	1 (7.1)	0.116
Cranial neuropathy, n (%)	0	0	0
Motor deficit, n (%)	0	0	0
Sensory deficit, n (%)	1 (6.2)	3 (21.4)	0.467
Ataxia, n (%)	2 (12.5)	1 (7.1)	0.552

All patients underwent cranial MRI examination at admission. MRI was within normal limits in only three (10%) patients. Diffuse meningeal thickening and gadolinium enhancement were seen in 24 (80%) patients, subdural collection (hematoma and/or hygroma) in 21 (70%) patients, pituitary gland engorgement in 14 (46.7%) patients, inferior displacement of the midbrain in 11 (36.7%) patients, prominence/dilation of venous sinuses in seven (23.3%) patients, cerebellar tonsillar descent in eight (26.7%) patients, medial and inferior migration of medial temporal lobes in five (16.7%) patients. The imaging findings of 17 patients with first-month MRI follow-up and 15 patients with sixth-month MRI follow-up are presented in Table 3. In only one of the 21 patients with first and/or sixth-month MRI follow-up, venous sinus prominence due to intracranial hypotension persisted.

**Table 3:** MRI findings of patients with intracranial hypotension at presentation and at first and sixth-month follow-up A significance level is p<0.05. MRI, magnetic resonance imaging

Cranial MRI findings	MRI at first admission (n=30)	First-month MRI follow-up (n=17)	Sixth-month MRI follow-up (n=15)
Normal, n (%)	3 (10)	7 (41.1)	14 (93.3)
Meningeal thickening/ enhancement, n (%)	24 (80)	9 (52.9)	0
Subdural collections, n (%)	21 (70)	6 (35.2)	0
Engorgement of the pituitary gland, n (%)	14 (46.7)	1 (5.8)	0
Inferior displacement of the midbrain, n (%)	11 (36.7)	1 (5.8)	0
Prominence of the venous sinuses, n (%)	7 (23.3)	1 (5.8)	1 (6.66)
Cerebellar tonsillar descent, n (%)	8 (26.7)	0	0
Migration of the medial temporal lobes, n (%)	5 (16.7)	0	0
Spinal MRI Findings			
Epidural fluid collection, n (%)	9 (30)	n/a	n/a
Epidural venous distention, n (%)	4 (13.3)	n/a	n/a

When MRI findings on admission were compared in patients with intracranial hypotension due to secondary causes and SIH patients, no significant difference was found between the frequency of findings seen on MRI (p>0.05) (Table 4). There was also no significant difference in the improvement rates of imaging findings in the 1st and 6th-month follow-up MRIs in patients with SIH and patients with intracranial hypotension due to secondary causes (p=0.788).

**Table 4:** MRI findings at presentation in patients with SIH and CSF leakage due to secondary causes A significance level is p<0.05. ^a^Mann-Whitney U Test MRI, magnetic resonance imaging; SIH, spontaneous intracranial hypotension; CSF, cerebrospinal fluid

Cranial MRI findings	SIH (n=16)	Secondary CSF leaks (n=14)	p^a^
Normal, n (%)	1 (6.3)	2 (14.3)	0.728
Meningeal thickening/enhancement, n (%)	14 (87.5)	10 (74.1)	0.637
Subdural collections, n (%)	12 (75)	9 (64.3)	0.473
Engorgement of the pituitary gland, n (%)	9 (56.3)	5 (35.7)	0.355
Inferior displacement of the midbrain, n (%)	5 (31.3)	6 (42.9)	0.608
Prominence of the venous sinuses, n (%)	3(18.8)	4 (28.6)	0.667
Cerebellar tonsillar descent, n (%)	4 (25)	4 (28.6)	0.886
Migration of the medial temporal lobes, n (%)	2 (12.5)	3 (21.4)	0.697

Twenty-one patients underwent spinal MRI examination. The site of leakage was determined by spinal MRI in nine (30%) patients. Nine (30%) patients had epidural fluid collection and four (13.3%) had epidural venous dilatation. A total of 11 patients with CSF leakage due to secondary causes (operation, LP, and spinal anesthesia) were not further investigated because the leakage site was clear. One patient underwent radionuclide cisternography, and one patient underwent MR myelography. Among patients with SIH or intracranial hypotension due to secondary causes, CSF leakage was detected in the cervical region in one patient and the thoracic region in six patients. Two patients had cervical+thoracic, one had thoracic+lumbar, and one had cervical+thoracic+lumbar CSF leak. In eight patients, the CSF leakage site could not be detected.

Bed rest, intravenous hydration, caffeine consumption, and analgesic treatment were applied to 19 (63.3%) patients, and EBP was administered to 11 (36.6) patients. EBP was administered once in seven patients, twice in two patients, three times in one patient, and four times in one patient. None of the patients required operation. Of the 11 patients who underwent EBP, nine had follow-up MRIs. Of the 19 patients who did not undergo EBP, 12 had follow-up MRIs. MRI findings at admission and at the first and sixth-month follow-up of patients who underwent and did not undergo EBP are presented in Table 5. There was no significant difference between the improvement rates of imaging findings in the first and sixth-month follow-up MRIs of patients treated with EBP or a conservative approach (p=0.788).

**Table 5:** MRI findings of patients who underwent EBP or were treated with conservative methods at presentation and at first and sixth-month follow-up A significance level is p<0.05. MRI, magnetic resonance imaging; EBP, epidural blood patch

Cranial MRI findings	Patients underwent EBP (n=11)			Patients treated with conservative approach (n=19)		
	MRI at first admission (n=11)	First-month MRI follow-up (n=7)	Sixth-month MRI follow-up (n=5)	MRI at first admission (n=19)	First-month MRI follow-up (n=10)	Sixth-month MRI follow-up (n=10)
Normal, n (%)	0 (0)	2 (28.5)	4 (80)	3 (15.8)	5 (50)	10 (100)
Meningeal thickening/enhancement, n (%)	11 (100)	5 (71.4)	0 (0)	13 (68.4)	4 (40)	0 (0)
Subdural collections, n (%)	11 (100)	2 (28.5)	1 (20)	10 (52.6)	4 (40)	0 (0)
Engorgement of the pituitary gland, n (%)	6 (54.5)	0 (0)	0 (0)	8 (42.1)	1 (10)	0 (0)
Inferior displacement of the midbrain, n (%)	5 (45.5)	0 (0)	0 (0)	6 (31.6)	1 (10)	0 (0)
Prominence of the venous sinuses, n (%)	2 (18.2)	1 (14.2)	0 (0)	5 (26.3)	0	0 (0)
Cerebellar tonsillar descent, n (%)	4 (36.4)	0 (0)	0 (0)	4 (21.1)	0	0 (0)
Migration of the medial temporal lobes, n (%)	3 (27.3)	0 (0)	0 (0)	2 (10.5)	0	0 (0)

## Discussion

One of the conditions that cause intracranial hypotension as a result of CSF leakage is SIH, which is a rare but well-known cause. In patients with SIH, CSF has somehow found an escape route and leaked out. CSF appears to leak mainly through a defect in the dura mater [15]. In our study, 53.3% of intracranial hypotension patients admitted in the last six years were diagnosed with SIH, while the rest had an identifiable cause that could explain CSF leakage. SIH was observed to be more common than CSF leakage due to secondary causes.

Intracranial hypotension headaches, as classified by the International Classification of Headache Disorders, 3rd edition, belong to the kind of secondary headache that results from a nonvascular intracranial disorder characterized by low CSF pressure [14]. It can be divided into three main groups. The first type is the post-dural puncture headache. The second type is the CSF fistula headache, and the third is the SIH-related headache. The typical symptom of intracranial hypotension is an orthostatic headache that worsens in a standing or sitting position. These symptoms may occur within a few seconds or take several hours to appear. It may disappear in less than a minute after moving to a supine position, but it can also last for hours [7,12,16]. Headaches usually range in intensity from pretty gentle to nearly unbearable [17]. The present study showed that 83.3% of intracranial hypotension patients had a postural headache. In our study, the incidence of headache was similar in patients with SIH and intracranial hypotension due to secondary causes. In contrast, posterior neck and shoulder pain were significantly higher in SIH patients. The fact that neck and shoulder pain are not common in iatrogenic and traumatic CSF leaks and are more common in SIH patients may be due to early diagnosis and symptomatic treatment, as patients in the secondary group are generally in the hospital at the time of headache onset or are admitted to the hospital early.

A patient's history, combined with MRI results, can lead to suspicion of intracranial hypotension. An MRI can confirm the diagnosis of a CSF leak. It is usually characterized by subdural fluid collections, typically bilateral and symmetrical. Fluid collections can also cause the brain to be pushed down from its normal position, a condition known as cerebral descent. Another finding on MRI that may indicate a CSF leak is an enlarged pituitary gland (usually filled with fluid from the leak). Venous structures draining the brain may also appear engorged [18,19]. However, 19% of people with CSF leakage may not show any abnormality on MRI [17]. In the present study, meningeal thickening and gadolinium enhancement were the most common findings in patients with intracranial hypotension, and subdural collections (hematoma and/or hygroma) were the second most common. There was no significant difference between the frequency of cranial MRI findings in patients with SIH and patients with intracranial hypotension due to secondary causes.

In patients with intracranial hypotension diagnosed by cranial MRI and/or lumbar puncture, the next step is to find out exactly where the patient's spinal fluid is leaking. The leakage is usually found in the spinal canal [18]. Methods such as CT myelography, dynamic myelography, and MR myelography with intrathecal gadolinium can accurately locate the CSF leak [20]. In the present study, spinal MRI was sufficient to localize the leak in nine of 21 patients. Further investigations were not required in patients with rapid clinical improvement. Only two patients underwent imaging other than spinal MRI to determine the leakage site. In most of the patients, follow-up spinal MRIs were not performed because clinical improvement started during the hospitalization phase and headache complaints regressed.

In our study, the majority of patients (63.3%) were managed conservatively. Conservative management is the usual initial treatment plan for CSF leaks. Bed rest and increased fluid intake are vital components, with some patients needing an even higher level of caffeine. Conservative management is successful for many individuals with intracranial hypotension [21]. However, it is not always effective, and some individuals may need more aggressive treatment [22]. Another method used to treat CSF leaking is an EBP. The EBP is an invasive procedure where the patient's blood is injected into the epidural space. The goal is to create a clot that forms over the defect in the dura mater, a kind of natural "band-aid" or "patch" made from the patient's blood. Many studies have presented EBP as a potentially superior alternative to conventional methods in certain patient groups [23]. Single case and multiple case studies have supported claims that EBP has success rates as high as 90% [21]. The risks associated with the procedure are mainly due to the potential for complications such as infections, hemorrhagia, or neurological deficits. EPB can lead to a dramatic reduction or complete resolution of the patient's symptoms, often within hours or days. Indeed, this rapid resolution distinguishes EPB from other potential treatments that can be quite time-consuming for both physician and patient and may not be as effective in the long term [24]. Although EBP is often successful, surgery may be necessary in some cases. Procedures such as endoscopic repair of a skull defect, suturing a tear in the dura, or reinforcing the dura with a graft are the most common procedures performed during surgical repair of a CSF leak. These surgeries may be recommended when conservative methods and EBP are insufficient or when the problem causing the CSF leak requires more attention [22,25]. In the present study, EBP was performed in 11 of 22 patients whose CSF leakage site was detected by spinal imaging or whose CSF leakage site was previously known in iatrogenic intracranial hypotension. However, there was no significant difference between the improvement rates of imaging findings in the follow-up MRIs of patients treated with EBP or a conservative approach. Surgical treatment was not required in any patient.

A major limitation of this study is the small sample size due to the rarity of intracranial hypotension. Another limitation is that in most patients, MRI or CT myelography was not performed because the leakage area was determined by contrast-enhanced spinal MRI.

## Conclusions

In conclusion, more significant posterior neck and shoulder pain was observed in SIH patients than in secondary intracranial hypotension patients. The fact that SIH patients are diagnosed later than secondary hypotension patients or that the leak site is often in the spinal cord in SIH patients may explain why neck and shoulder pain are more pronounced in these patients.

Furthermore, according to our results, cranial MRI features and radiologic recovery times are similar in patients with intracranial hypotension regardless of etiology. Again, no difference was found between the radiologic recovery times in patients who underwent conservative treatment and EBP. Treatment approaches and recovery times for intracranial hypotension due to spontaneous or secondary causes are similar. These findings should be evaluated in larger study groups.
